# *Bacillus kwashiorkori* sp. nov., a new bacterial species isolated from a malnourished child using culturomics

**DOI:** 10.1002/mbo3.535

**Published:** 2017-10-27

**Authors:** El hadji Seck, Mamadou Beye, Sory Ibrahima Traore, Saber Khelaifia, Caroline Michelle, Carine Couderc, Souleymane Brah, Pierre‐Edouard Fournier, Didier Raoult, Fadi Bittar

**Affiliations:** ^1^ Aix‐Marseille Univ CNRS 7278 AP‐HM IRD 198, INSERM 1095 URMITE IHU Méditerranée Infection Marseille France; ^2^ Hôpital National de Niamey Niamey Niger; ^3^ King Fahd Medical Research Center Special Infectious Agents Unit King Abdulaziz University Jeddah Saudi Arabia

**Keywords:** *Bacillus kwashiorkori*, culturomics, genome, taxonogenomics

## Abstract

Strain SIT6^T^ was isolated from the fecal flora of a severely malnourished child as part of a broad “culturomics” study aiming to maximize the culture conditions for the in‐depth exploration of the human microbiota. An analysis of the 16S rRNA gene sequence showed that strain SIT6^T^ shared 94.1% 16S rRNA gene sequence similarity with *Bacillus thermoamylovorans* DKP^T^ (NR_029151), the phylogenetically closest type species. Colonies are creamy white, circular, 4–5 mm in diameter after cultivation at 37°C for 24 hr on 5% sheep blood‐enriched Colombia agar. Growth occurs at temperatures in the range of 25–56°C (optimally at 37°C). Strain SIT6^T^ is a gram‐positive, facultative anaerobic rod and motile by means of peritrichous flagella and sporulating; it is catalase and oxidase positive. The 2,784,637‐bp‐long genome, composed of 16 contigs, has a G+C content of 35.19%. Of the 2,646 predicted genes, 2,572 were protein‐coding genes and 74 were RNAs. The major fatty acids are saturated species (15:0 iso, 16:0 and 17:0 anteiso). Of the 14 detected fatty acids, 11 are saturated, either linear or branched (iso and anteiso). Digital DNA–DNA hybridization (dDDH) estimation and average genomic identity of orthologous gene sequences (AGIOS) of the strain SIT6^T^ against genomes of the type strains of related species ranged between 18.6% and 38.3% and between 54.77% and 65.50%, respectively. According to our taxonogenomics results, we propose the creation of *Bacillus kwashiorkori* sp. nov. that contains the type strain SIT6^T^ (=CSUR P2452^T^, =DSM 29059^T^).

## 1 INTRODUCTION

Although the human intestinal flora is intrinsically associated with the host genotype and age, many external factors can affect and modify this microbiota, such as antibiotics, probiotics, and diet (Angelakis, Armougom, Million, & Raoult, 2012; Chen, He, & Huang, 2014; Moreno‐Indias, Cardona, Tinahones, & Queipo‐Ortuño, 2014). Recently, genomic and metagenomic advances have widely participated in describing the human microbiota, but culture isolation remains the only means and the first step to characterize the physiological and genomic properties of a given bacterium and to describe a potential new species (Vartoukian, Palmer, & Wade, 2010). For this reason, in our laboratory we have developed a new strategy called culturomics, which is based on the application of various culture conditions followed by rapid identification using matrix‐assisted laser‐desorption/ionization time‐of‐flight mass spectrometry (MALDI‐TOF MS) to explore the bacterial composition (Lagier et al., 2012). This new concept has allowed us to significantly increase the bacterial species associated with the human digestive tract and to find many new species (Lagier et al., 2016). Using this strategy (i.e., culturomics), we were able to isolate a new species belonging to the genus *Bacillus*.

This new isolate was described according to the new method that we have implemented (taxonogenomics) (Kokcha et al., 2012; Lagier, Elkarkouri, Rivet, Couderc, & Raoult, 2013; Seck et al., 2016). In brief, it involves using proteomic, fatty acid, and genomic features (Ramasamy et al., 2014; Welker & Moore, 2011; Seng et al., 2013), along with phenotype and some conventional methods, such as 16S rRNA phylogeny and the G+C content.

In this article, we describe the strain SIT6^T^ (=CSUR P2452^T^, =DSM 29059^T^) isolated from the stool sample of a kwashiorkor patient.

## 2 MATERIALS AND METHODS

### 2.1 Organism information

The study and consent procedure were approved by the National Ethics Committee of Nigeria and the Ethics Committee of the Federative Research Institute 48 (Faculty of Medicine, Marseille, France) under the agreement number 09‐022. The stool sample was obtained from a 4‐month‐old Nigerian child suffering from acute malnutrition (kwashiorkor). The patient was not being treated with antibiotics at the time of the sample collection and the sample was stored at −80°C. The stool sample was cultured in blood culture bottles supplemented with sheep blood (BioMérieux, Marcy l'Etoile, France). During a 30‐day preincubation period at 37°C in aerobic atmosphere, the liquid culture is then spread on Columbia agar with 5% sheep blood COS medium (BioMérieux, Marcy l'Etoile, France) and the isolated colonies are subsequently identified.

### 2.2 Strain identification by MALDI‐TOF MS and 16S rRNA sequencing

MALDI‐TOF MS analysis of proteins was used to identify the bacteria. Each colony was deposited in duplicate on a MALDI‐TOF MSP 96 target and then covered with 1.5 μl of a matrix solution (saturated solution of α‐cyano‐4‐hydroxycinnamic acid in 50% acetonitrile, 2.5% trifluoroacetic acid) to allow the crystallization of molecules. MALDI‐TOF MS was performed using the LT Microflex spectrometer (Bruker Daltonics, Leipzig, Germany). All spectra were recorded in positive linear mode for the mass range from 2,000 to 20,000 Da (parameters: ion source 1 [ISI], 20 kV; IS2, 18.5 kV lens, 7 kV). The generated spectra were then compared to the Bruker database, with the addition of new species found through the “culturomics” project. The resulting score dictates whether a tested species can be identified: a score ≥2 with a validly published species enables identification at the species level, a score ≥1.7 but <2 enables identification at the genus level, and a score <1.7 does not enable any identification.

Following three assays, unidentified colonies were identified using 16S rRNA gene sequencing as described previously (Bittar et al., 2014). The isolated colony was suspended in 200 μl distilled water for DNA extraction using an EZ1 DNA Tissue Kit with a BioRobot EZ1 Advanced XL (Qiagen, Courtaboeuf, France). The amplification of the 16S rRNA gene was performed using the universal primer pair fD1 and rP2 (Eurogentec, Angers, France) (Weisburg, Barns, Pelletier, & Lane, 1991). The PCR product was purified and sequenced using the BigDye Terminator v1.1 Cycle Sequencing Kit (PerkinElmer, Courtaboeuf, France) with the following internal primers: 536F, 536R, 800F, 800R, 1050F, and 1050R, and ABI Prism 3130xl Genetic Analyzer capillary sequencer (Applied Biosystems). 16S rRNA amplification and sequencing were carried out as described previously by Morel et al. (2015). The 16S rRNA nucleotide sequences were assembled and corrected using CodonCode Aligner software (http://www.codoncode.com). Then, the BLASTn searches against the GenBank NCBI database (http://blast.ncbi.nlm.nih.gov.gate1.inist.fr/Blast.cgi) and EzBioCloud's Identify Service (http://www.ezbiocloud.net/identify) (Yoon et al., 2017) were performed to determine the percentage of similarity with the closest bacteria.

The MEGA 7 (Molecular Evolutionary Genetics Analysis) software (Kumar, Stecher, & Tamura, 2016) allowed us to construct a phylogenetic tree. Sequence alignment of the different species was performed using CLUSTALW and the calculation of the evolutionary distance was done with the Kimura two‐parameter model (Kimura, 1980; Thompson, Higgins, & Gibson, 1994).

### 2.3 Growth conditions

In order to determine the ideal growth conditions for strain SIT6^T^, different growth temperatures (25°C, 28°C, 30°C, 37°C, 45°C, and 56°C) were tested under anaerobic and microaerophilic atmospheres using GENbag Anaer and GENbag microaer systems, respectively (BioMérieux, Marcy l'Etoile, France). The strain growth was also tested aerobically with and without 5% CO_2_. The growth of strain SIT6^T^ was tested under different pH using a Columbia agar with 5% sheep blood COS medium (BioMérieux, Marcy l'Etoile, France) with NaCl, MgCl_2_, MgSO_4_, KCl, CaCl_2_, and glucose. The pH was modified by adding HCl to the medium and measured with a pH meter. The optimal pH for growth was determined by testing at different pH 5, 6, 6.5, 7, 7.5, 8, and 8.5. Growth at various NaCl concentrations (0.5%, 5%, 7.5%, 10%, 15%, and 200%) was investigated.

### 2.4 Morphologic, biochemical, and antibiotic susceptibility tests

Gram staining was performed and observed using a Leica DM 2500 photonic microscope (Leica Microsystems, Nanterre, France) with a 100× oil immersion lens. A thermal shock (80°C during 20 min) was applied on fresh colonies in order to test sporulation. The motility of the strain was tested by observing fresh colonies using a DM1000 photonic microscope (Leica Microsystems) with a 40× objective lens. Catalase (BioMérieux) activity was determined in 3% hydrogen peroxide solution and oxidase activity was assessed using an oxidase reagent (Becton‐Dickinson, Le Pont‐de‐Claix, France).

Antibiotic susceptibility testing was performed using SIRscan Discs (i2a, Montpellier, France) on Mueller‐Hinton agar according to EUCAST 2015 recommendations (Matuschek, Brown, & Kahlmeter, 2014). The following antibiotics were tested: doxycycline (30 μg), rifampicin (30 μg), vancomycin (30 μg), erythromycin (15 μg), ampicillin (10 μg), ceftriaxone (30 μg), ciprofloxacin (5 μg), gentamicin (500 μg), penicillin (10 μg), trimethoprim/sulfamethoxazole (25 + 23.75 μg), imipenem (10 μg), metronidazole (4 μg), clindamycin (15 μg), colistin (50 μg), and oxacillin (5 μg).

Using the commercially available biochemical API 20NE, API ZYM, and API 50CH strips, we investigated the biochemical characteristics of our strain according to the manufacturer's instructions (BioMérieux).

Negative staining was done in order to visualize the cell morphology. Cells were fixed with 2.5% glutaraldehyde in 0.1 mol/L cacodylate buffer for at least 1 hr at 4°C. A drop of cell suspension was deposited for approximately 5 min on glow‐discharged formvar carbon film on 400 mesh nickel grids (FCF400‐Ni, EMS). The grids were dried on blotting paper and the cells were negatively stained for 10 s with 1% ammonium molybdate solution in filtered water at room temperature. Electron micrographs were acquired with a Tecnai G20 Cryo (FEI) transmission electron microscope operated at 200 keV.

### 2.5 FAME analysis by gas chromatography/mass spectrometry

Cellular fatty acid methyl ester (FAME) analysis was performed by GC/MS. Two samples were prepared with approximately 2 mg of bacterial biomass each, harvested from five culture plates. Fatty acid methyl esters were prepared as described by Sasser (2006). GC/MS analyses were carried out as described previously (Dione et al., 2016). In brief, fatty acid methyl esters were separated using an Elite 5‐MS column and monitored by mass spectrometry (MS) (Clarus 500‐SQ 8 S, PerkinElmer, Courtaboeuf, France). A spectral database search was performed using MS Search 2.0 operated with the Standard Reference Database 1A (NIST, Gaithersburg, MD, USA) and the FAMEs mass spectral database (Wiley, Chichester, UK).

### 2.6 Genomic DNA preparation

After pretreatment by a lysozyme (incubation at 37°C for 2 hr), the DNA of strain SIT6^T^ was extracted on the EZ1 BioRobot (Qiagen) with the EZ1 DNA tissues kit. The elution volume was 50 μl. Genomic DNA (gDNA) was quantified by a Qubit assay with the high sensitivity kit (Life Technologies, Carlsbad, CA) to 55.8 ng/μl.

### 2.7 Genome sequencing and assembly

Genomic DNA (gDNA) of *B. kwashiorkori* was sequenced on MiSeq Technology (Illumina Inc., San Diego, CA) with the mate pair strategy. The gDNA was barcoded in order to be mixed with 11 other projects with the Nextera Mate Pair sample prep kit (Illumina). gDNA was quantified by a Qubit assay with the high sensitivity kit (Thermo Fisher Scientific, Waltham, MA) to 66.2 ng/μl. The mate pair library was prepared with 1 μg of gDNA using the Nextera mate pair Illumina guide. The gDNA sample was simultaneously fragmented and tagged with a mate pair junction adapter. The pattern of the fragmentation was validated on an Agilent 2100 Bioanalyzer (Agilent Technologies Inc., Santa Clara, CA) with a DNA 7500 LabChip. The DNA fragments ranged in size from 1 kb to 11 kb, with an optimal size at 3.927 kb. No size selection was performed and 505 ng of tagged fragments were circularized. The circularized DNA was mechanically sheared to small fragments with an optimal size of 597 bp on the Covaris device S2 in microtubes (Covaris, Woburn, MA). The library profile was viewed on a High Sensitivity Bioanalyzer LabChip (Agilent Technologies Inc.) and the final concentration library was measured at 59.2 nmol/L. The libraries were normalized at 2 nmol/L and pooled. After a denaturation step and dilution at 15 pmol/L, the pool of libraries was loaded onto the reagent cartridge and then onto the instrument along with the flow cell. An automated cluster generation and sequencing run was performed in a single 39‐hr run in a 2 × 251 bp.

### 2.8 Genome annotation and analysis

Open reading frames (ORFs) were predicted using Prodigal (http://prodigal.ornl.gov/) with default parameters. However, the predicted ORFs were excluded if they spanned a sequencing gap region. The predicted bacterial protein sequences were searched against GenBank and Clusters of Orthologous Group (COG) databases using BLASTP. The tRNAs and rRNAs were predicted using the tRNAScan‐SE and RNAmmer tools, respectively. Signal peptides and numbers of transmembrane helices were predicted using SignalP (Nielsen, Engelbrecht, Brunak, & von Heijne, 1997) and TMHMM (Krogh, Larsson, von Heijne, & Sonnhammer, 2001), respectively. Mobile genetic elements were predicted using PHAST (Zhou, Liang, Lynch, Dennis, & Wishart, 2011) and RAST (Aziz et al., 2008). ORFans were identified if their BLASTP E‐value was lower than 1e‐03 for alignment length >80 amino acids. If alignment lengths were <80 amino acids, we used an E‐value of 1e‐05. Such parameter thresholds have already been used in previous work to define ORFans. Artemis (Carver, Harris, Berriman, Parkhill, & McQuillan, 2012) and DNA plotter (Carver, Thomson, Bleasby, Berriman, & Parkhill, 2009) were used for data management and visualization of genomic features, respectively. The mauve alignment tool (version 2.3.1) was used for multiple genomic sequence alignment (Darling, Mau, Blattner, & Perna, 2004). The mean level of nucleotide sequence similarity at the genome level between *B. kwashiorkori* and other *Bacillus* species was estimated using the Average Genomic Identity of Orthologous Gene Sequences (AGIOS) in‐house software (Ramasamy et al., 2014). This software combines the functionality of other software programs: Proteinortho (Lechner et al., 2011) (detects orthologous proteins between genomes compared two by two, then retrieves the corresponding genes) and the Needleman–Wunsch global alignment algorithm (determines the mean percentage of nucleotide sequence identity among orthologous ORFs). Finally, the Genome‐to‐Genome Distance Calculator (GGDC) web server (http://ggdc.dsmz.de) was used to estimate the similarity between the compared genomes (Auch, Jan, Klenk, & Göker, 2010; Meier‐Kolthoff, Auch, Klenk, & Göker, 2013).

Average nucleotide identity at the genome level between *B. kwashiorkori* (CTDX00000000) and the other species *B. firmus* (BCUY00000000), *B. shackletonii* (LJJC00000000), *B. smithii* (BCVY00000000), *B. aquimaris* (LQXM00000000), *B. thermoamylovorans* (CCRF00000000), *B. coagulans* (CP003056), *B. alveayuensis* (JYCE00000000), *B. sporothermodurans* (LQYN00000000), *B. acidicola* (LWJG00000000), and *B. ginsengihumi* (JAGM00000000) was estimated using BLASTN and a home made software, following the algorithm described by Ouk, Chun, Lee, and Park (2016).

## 3 RESULTS AND DISCUSSION

### 3.1 Strain identification and phylogenetic analyses

**Figure 1:**
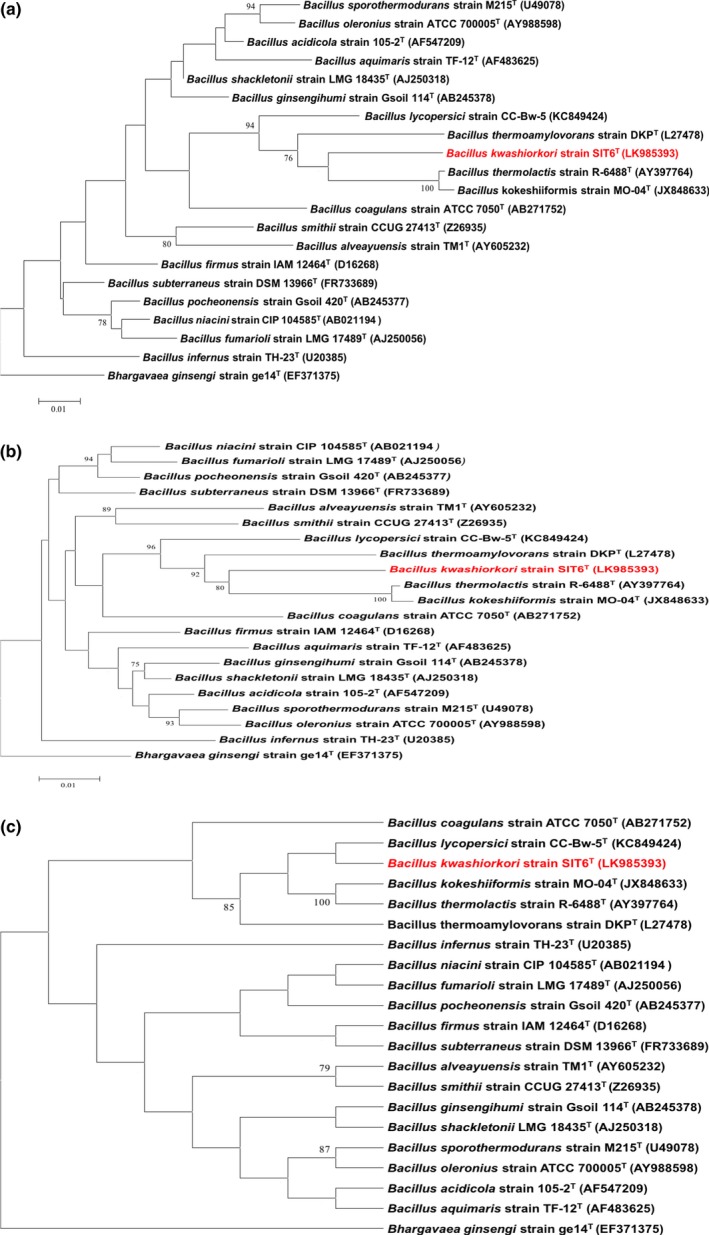
Phylogenetic tree showing the position of *Bacillus kwashiorkori* SIT6^T^ (red) relative to other phylogenetically close members of the family *Bacillaceae*. GenBank accession numbers are indicated in parentheses. Sequences were aligned using CLUSTALW, and phylogenetic inferences were obtained using (a) the maximum‐likelihood method, (b) the neighbor‐joining method and (c) the maximum parsimony method within the MEGA software. Numbers at the nodes are percentages of bootstrap values obtained by repeating the analysis 1,000 times to generate a majority consensus tree. Only values >70% were displayed. *Bhargavaea ginsengi* ge14^T^ (EF371375) was used as out‐group

**Figure 2:**
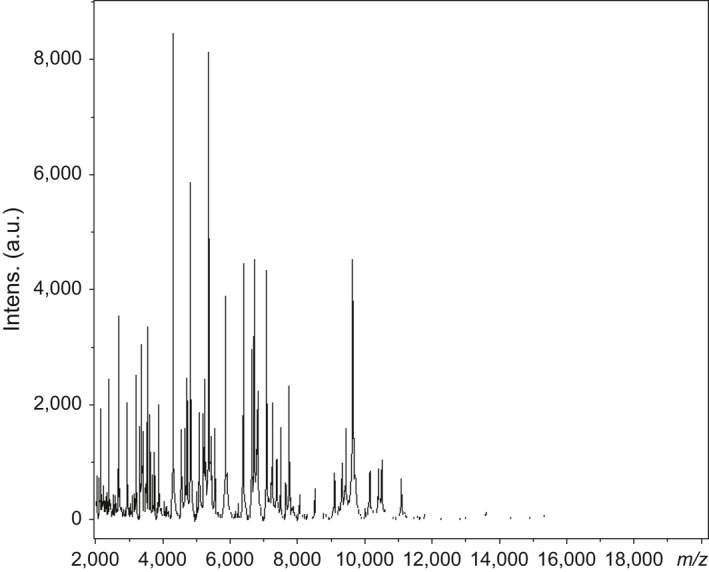
Reference mass spectrum from *Bacillus kwashiorkori* SIT6^T^ strain. Spectra from 12 individual colonies were compared and a reference spectrum was generated

**Figure 3:**
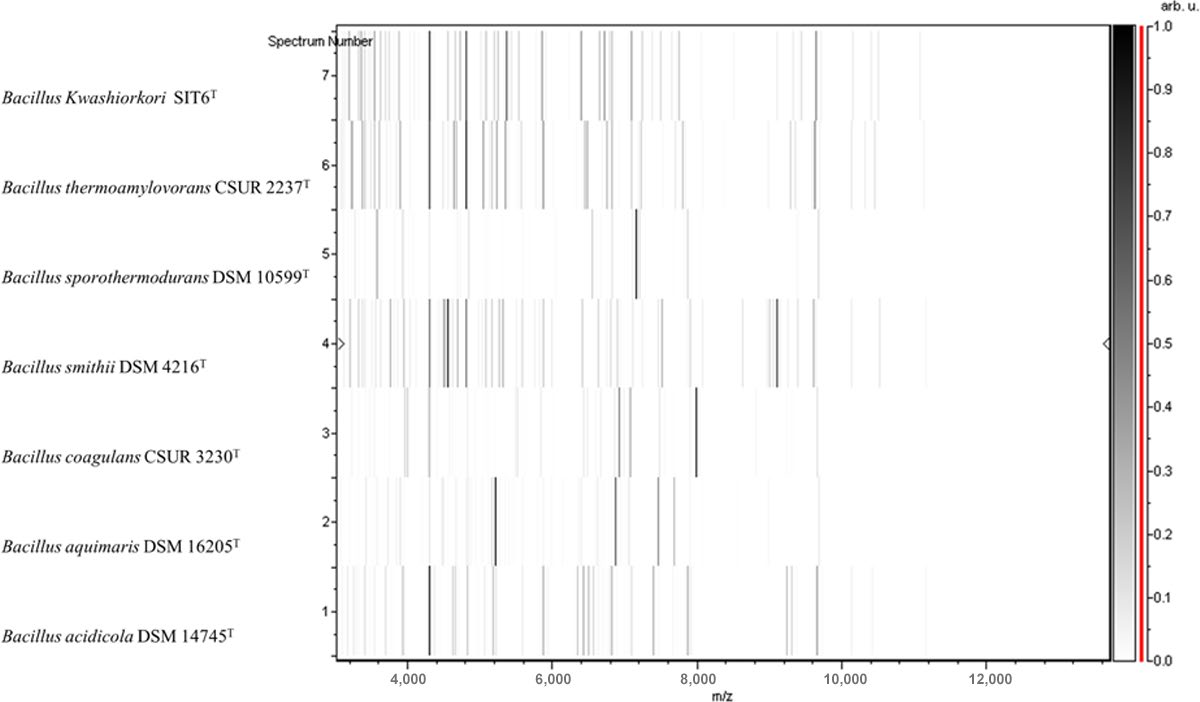
Gel view comparing *Bacillus kwashiorkori* SIT6^T^ spectra with other members of the genus *Bacillus*. The gel view displays the raw spectra of all loaded spectrum files arranged in a pseudo‐gel‐like look. The *x*‐axis records the m/z value. The left *y*‐axis displays the running spectrum number originating from subsequent spectra loading. The peak intensity is expressed by a gray scale scheme code. The color bar and the right *y*‐axis indicate the relation between the color a peak is displayed with and the peak intensity in arbitrary units. Displayed species are indicated on the right

Strain SIT6^T^ was first isolated in May 2014 after a 30‐day preincubation in a blood culture bottle with sheep blood and cultivation on 5% sheep blood‐enriched Colombia agar in an aerobic atmosphere at 37°C. No significant MALDI‐TOF score was obtained for strain SIT6^T^ against the Bruker and URMITE databases, suggesting that the isolate was not a member of a known species. Strain SIT6^T^ shared 94.1% 16S rRNA gene sequence similarity with *B. thermoamylovorans* DKP^T^ (NR_029151) using GenBank NCBI database (reference RNA sequences). Although the 16S rRNA gene sequence of strain SIT6^T^ showed 94.58% similarity with *Bacillus kokeshiiformis* MO‐04^T^ and 94.57% similarity with *Bacillus thermolactis* R‐6488^T^ by EzBioCloud's identify server. Figure 1a, b, and c present the phylogenetic trees of strain SIT6^T^ relative to other closest type species with a validly published name using maximum‐likelihood, neighbor‐joining, and maximum parsimony methods, respectively. Consequently, as this 16S rRNA nucleotide sequence similarity was lower than the threshold of 98% recommended by Tindall, Rosselló‐Mora, Busse, Ludwig, and Kämpfer (2010) to delineate a new species; it was classified as a new species called *Bacillus kwashiorkori* SIT6^T^ (Table 1). Furthermore, this percentage of similarity comprised in the range of percentage similarity of *Bacillus* species (82.7–100%), confirming the new species status (Rossi‐Tamisier, Benamar, Raoult, & Fournier, 2015). The reference spectrum for strain SIT6^T^ was thus incremented in the URMITE database (http://www.mediterranee-infection.com/article.php?laref=256&titre=urms-database) (Figure 2) and then compared to other known species of the genus *Bacillus*. The differences exhibited are shown in the obtained gel view (Figure 3).

**Table 1:** Classification and general features of *Bacillus kwashiorkori* strain SIT6^T^

Property	Term
Current classification	Domain: Bacteria
	Phylum: Firmicutes
	Class: Bacilli
	Order: Bacillales
	Family: Bacillaceae
	Genus: *Bacillus*
	Species: *kwashiorkori*
	Type strain: SIT6^T^
Gram stain	Positive
Cell shape	Rod shaped
Motility	Motile
Sporulation	Endospore forming
Temperature range	Mesophile
Optimum temperature	37°C
Optimum pH	7.5
Salinity	0.0–5.0 g/L
Optimum salinity	0 g/L
Oxygen requirement	Facultative aerobic

### 3.2 Phenotypic description

**Figure 4:**
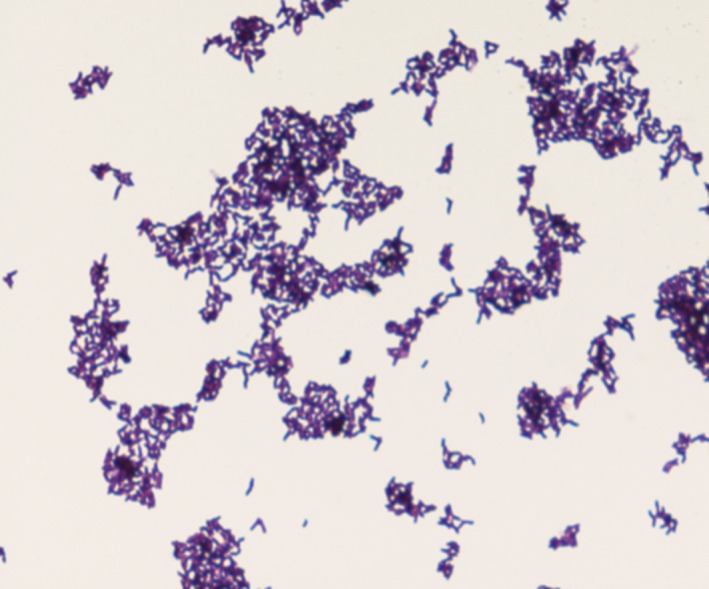
Gram staining of *Bacillus kwashiorkori* SIT6^T^

**Figure 5:**
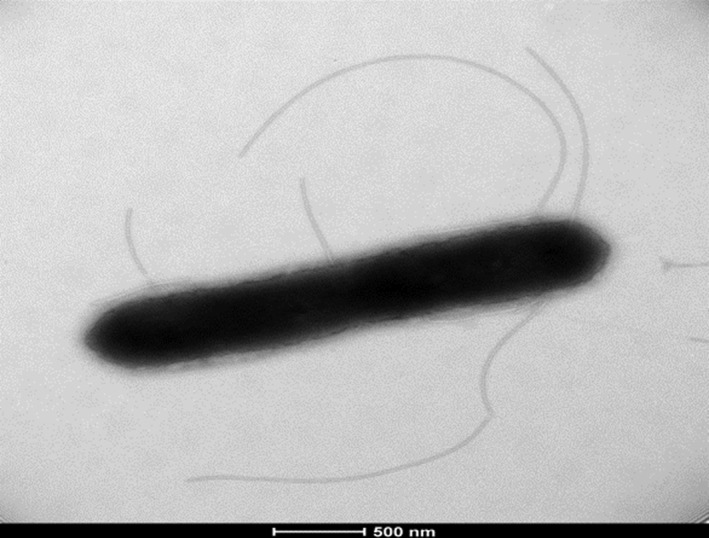
Transmission electron microscopy of *Bacillus kwashiorkori* SIT6^T^ using a Morgani 268D (Philips) at an operating voltage of 60 kV. The scale bar represents 500 nm

Growth of strain SIT6^T^ was observed between 25°C and 56°C on 5% sheep blood Colombia agar and optimal growth was achieved at 37°C after 24 hr incubation in aerobic conditions (37°C was the temperature at which this strain grows most rapidly). Poor growth occurred under microaerophilic and anaerobic conditions. Cells were motile and sporulating. Colonies were circular, white with a mean diameter of 5 mm on blood‐enriched Colombia agar. Gram staining (Figure 4) showed gram‐positive rods. Using electron microscopy, the rods had a mean diameter of 1.8 μm and a length of 5.9 μm (Figure 5). Catalase and oxidase activities were positive for strain SIT6^T^.

The major fatty acids are saturated species (15:0 iso, 16:0 and 17:0 anteiso). Of the 14 detected fatty acids, 11 are saturated, either linear or branched (iso and anteiso). The fatty acid composition of strain SIT6^T^ is detailed in Table 2.

**Table 2:** Cellular fatty acid composition

Fatty acids	IUPAC name	Mean relative %^a^
15:0 iso	13‐Methyl‐tetradecanoic acid	19.6 ± 1.2
16:0	Hexadecanoic acid	19.5 ± 0.4
17:0 anteiso	14‐Methyl‐hexadecanoic acid	16.5 ± 1.3
18:1n12	6‐Octadecenoic acid	12.7 ± 1.9
18:0	Octadecanoic acid	9.3 ± 0.4
17:0 iso	15‐Methyl‐hexadecanoic acid	6.9 ± 1.7
15:0 anteiso	12‐Methyl‐tetradecanoic acid	4.5 ± 0.1
18:2n6	9,12‐Octadecadienoic acid	4.0 ± 0.2
16:0 iso	14‐Methyl‐pentadecanoic acid	3.9 ± 0.5
18:1n5	13‐Octadecenoic acid	1.5 ± 0.1
14:0	Tetradecanoic acid	TR
17:0	Heptadecanoic acid	TR
15:0	Pentadecanoic acid	TR
14:0 iso	12‐Methyl‐tridecanoic acid	TR

a Mean peak area percentage ± standard deviation (*n* = 3); TR, trace amounts <1%.

Table 3 shows the biochemical features of *B. kwashiorkori* SIT6^T^ and the most closely related species.

**Table 3:** Phenotypic characteristics of *Bacillus kwashiorkori* SIT6^T^ with other phylogenetically close *Bacillus* strains^a^

Properties	*B. kwashiorkori*	*B. thermoamylovorans*	*B. thermolactis*	*B. smithii*	*B. aquimaris*	*B. acidicola*	*B. sporothermodurans*	*B. alveayuensis*	*B. coagulans*
Cell diameter (μm)	0.2–3.0	0.45–4.0	0.6–10	0.5–1.1	0.45–1	3.1–5.9	NA	0.7–4.5	0.6–1
Oxygen requirement	Facultative	Facultative	Facultative	Facultative	Aerobic	Facultative	Aerobic	Facultative	Facultative
	Anaerobic	Anaerobic	Anaerobic	Anaerobic		Anaerobic		Anaerobic	Anaerobic
Gram stain	+	+	+	+	v	+	+	+	+
Motility	+	−	−	+	+	NA	+	+	+
Endospore formation	+	+	+	+	+	+	+	+	+
Indole	−	−	−	−	NA	−	−	−	−
*Production of*									
Catalase	+	+	+	+	+	NA	+	NA	NA
Oxidase	+	−	+	+	−	−	+	−	NA
Nitrate reductase	−	+	+	−	+	NA	−	−	+
Urease	−	−	−	−	−	NA	−	−	−
d‐Galactosidase	+	+	−	−	+	NA	NA	−	+
*N*‐acetyl‐glucosamine	−	−	−	−	−	NA	NA	−	+
*N*‐acetyl‐β‐glucosaminidase	−	NA	NA	+	NA	NA	NA	NA	NA
Alpha‐mannosidase	+	NA	NA	NA	NA	NA	NA	NA	+
*Acid from*									
l‐Arabinose	−	−	+	+	NA	−	NA	−	+
Glycerol	−	−	−	+	−	+	NA	+	NA
Erythritol	−	NA	NA	+	NA	NA	NA	−	NA
d‐Ribose	−	+	+	+	NA	+	NA	−	−
d‐Xylose	−	−	+	+	−	+	−	−	−
d‐Adonitol	−	NA	NA	−	NA	NA	NA	−	NA
Methyl‐β‐d‐xylopyranose	−	NA	NA	−	NA	NA	NA	−	NA
l‐Sorbose	−	+	NA	−	NA	−	NA	NA	+
l‐Rhamnose	−	−	NA	+	NA	−	NA	NA	−
Dulcitol	−	NA	NA	−	NA	NA	NA	NA	+
Inositol	−	NA	NA	+	NA	NA	NA	NA	NA
d‐Sorbitol	−	NA	NA	+	NA	−	NA	NA	−
Esculin	+	NA	NA	+	NA	NA	NA	NA	NA
d‐Raffinose	+	−	−	NA	−	NA	−	−	NA
d‐Lyxose	−	NA	NA	−	NA	NA	NA	NA	NA
d‐Fucose	−	NA	NA	−	NA	NA	NA	NA	−
l‐Arabitol	−	NA	NA	NA	NA	−	NA	NA	−
d‐Mannose	+	−	−	+	−	+	−	+	+
d‐mannitol	+	−	v	+	−	+	−	−	−
d‐Glucose	+	+	−	+	+	+	+	+	+
d‐Fructose	+	+	−	+	+	+	−	+	+
d‐Maltose	+	−	+	+	+	+	+	−	+
d‐Lactose	−	−	−	+	−	v	−	−	+
Galactose	−	−	−	+	−	+	−	+	+
Habitat	Human gut	Oil	Milk	Milk	Sea	Acidic sphagnum	Water, milk	Sea	Milk

NA, not available; v, variable.

a *Bacillus thermoamylovorans* DKP^T^ (Combet‐Blanc et al., 1995), *Bacillus thermolactis* R‐6488^T^ (Coorevits et al., 2011), *Bacillus smithii* NRRL NRS‐173^T^ (Bae, Lee, & Kim, 2005), *Bacillus aquimaris* TF‐12^T^ (Yoon, Kim, Kang, Oh, & Park, 2003), *Bacillus sporothermodurans* M215^T^ (Heyndrickx et al., 2012), *Bacillus acidicola* 105‐2^T^ (Albert, 2005), *Bacillus alveayuensis* TM1^T^ (Bae et al., 2005), and *Bacillus coagulans* 2‐6^T^ (De Clerck et al., 2004).

Bacterial cells were resistant to metronidazole, but susceptible to imipenem, doxycycline, rifampicin, vancomycin, amoxicillin, ceftriaxone, gentamicin, trimethoprim/sulfamethoxazole, erythromycin, ciprofloxacin, and gentamicin.

### 3.3 Genome properties

**Figure 6:**
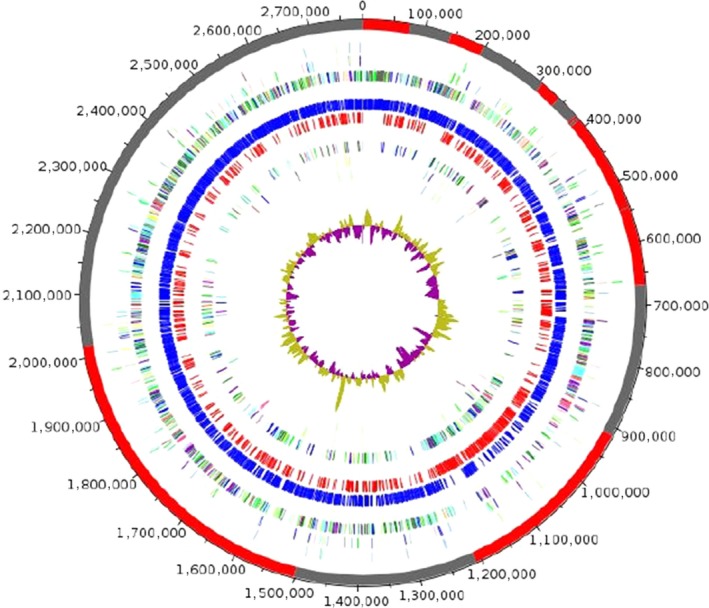
Graphical circular map of the chromosome. From outside to the center: Genes on the forward strand colored by clusters of orthologous groups (COG) categories (only gene assigned to COG), genes on the reverse strand colored by COG categories (only gene assigned to COG), RNA genes (tRNAs green, rRNAs red), G+C content and G+C skew

The genome is 2,784,637 bp long with 35.19% G+C content (Figure 6). It is composed of 16 scaffolds, composed of 16 contigs. Of the 2,646 predicted genes, 2,572 were protein‐coding genes, and 74 were RNAs (7 genes are 5S rRNA, 2 genes are 16S rRNA, 2 genes are 23S rRNA, and 63 genes are tRNA). A total of 1,749 (68%) were assigned as putative function (by COGs of NR blast). A total of 156 genes were identified as ORFans (6.07%). The remaining genes were annotated as hypothetical proteins (487 genes [18.93%]). Genome content is detailed in Table 4, while Table 5 presents the distribution of the genes into COG functional categories.

**Table 4:** Nucleotide content and gene count levels of the genome

Attribute	Value	% of total^a^
Genome size (bp)	2,784,637	100
DNA coding region (bp)	2,319,082	83.28
DNA G+C content (bp)	980,134	35.19
Total genes	2,646	100
RNA genes	74	2.79
tRNA genes	63	2.38
Protein‐coding genes	2,572	97.20
Genes with function prediction	1,749	68.00
Genes assigned to COGs	1,715	66.68
Protein associated with hypothetical protein	487	18.93
Protein associated with ORFan	156	6.06
Genes with peptide signals	250	9.72
Genes with transmembrane helices	720	27.99
Genes associated with PKS or NRPS	6	0.2
Genes associated with mobilome	1,402	54.51
Genes associated with toxin/antitoxin	84	3.26

a The total is based on either the size of the genome in base pairs or the total number of protein‐coding genes in the annotated genome.

**Table 5:** Number of genes associated with the 25 general COG functional categories

Code	Value	%^a^	Description
J	130	5.05	Translation
A	0	0	RNA processing and modification
K	141	5.48	Transcription
L	140	5.44	Replication, recombination, and repair
B	1	0.04	Chromatin structure and dynamics
D	18	0.70	Cell cycle control, mitosis, and meiosis
Y	0	0	Nuclear structure
V	43	1.67	Defense mechanisms
T	88	3.42	Signal transduction mechanisms
M	81	3.15	Cell wall/membrane biogenesis
N	26	1.01	Cell motility
Z	0	0	Cytoskeleton
W	0	0	Extracellular structures
U	26	1.01	Intracellular trafficking and secretion
O	87	3.38	Posttranslational modification, protein turnover, chaperones
C	120	4.67	Energy production and conversion
G	137	5.33	Carbohydrate transport and metabolism
E	138	5.37	Amino acid transport and metabolism
F	39	1.52	Nucleotide transport and metabolism
H	52	2.02	Coenzyme transport and metabolism
I	67	2.61	Lipid transport and metabolism
P	147	5.72	Inorganic ion transport and metabolism
Q	34	1.32	Secondary metabolites biosynthesis, transport, and catabolism
R	241	9.37	General function prediction only
S	180	6.99	Function unknown
−	857	33.32	Not in COGs

a The total is based on the total number of protein‐coding genes in the annotated genome.

The genome sequence has been deposited in GenBank under accession number CTDX00000000.

### 3.4 Comparison with other *Bacillus* spp. genomes

**Figure 7:**
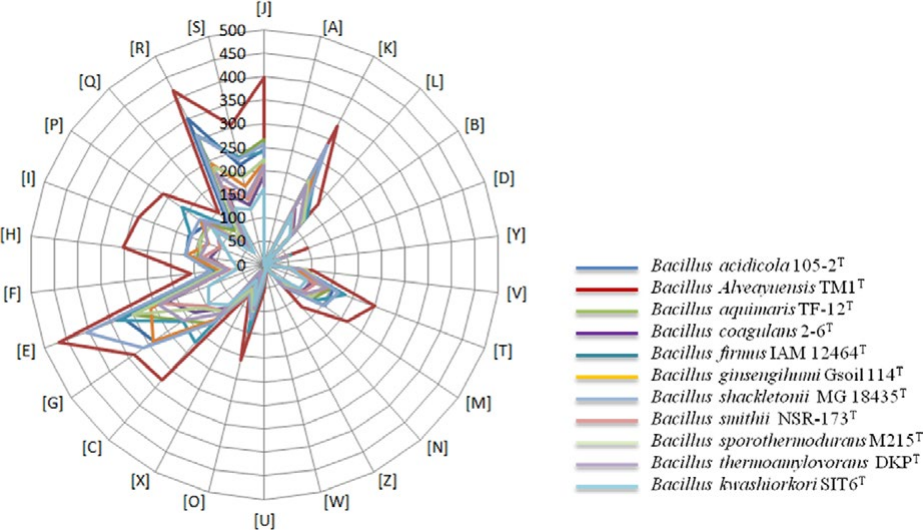
Distribution of functional classes of predicted genes of *Bacillus kwashiorkori* SIT6^T^ with 10 members of *Bacillus* genus

The draft genome of *B. kwashiorkori* (2.78 Mb) is smaller in size than those of *Bacillus alveayuensis*,* Bacillus shackletonii*,* Bacillus coagulans*,* Bacillus ginsengihumi*,* Bacillus firmus*,* Bacillus aquimaris*,* Bacillus sporothermodurans*,* Bacillus smithii*,* Bacillus acidicola*, and *Bacillus thermoamylovorans* (6.70, 5.29, 3.07, 3.92, 4.97, 4.42, 4.04, 3.38, 5.13, and 3.70 Mb, respectively) (Table 6). *Bacillus kwashiorkori* has a lower G+C content (35.19%) than those of *B. alveayuensis*,* B. shackletonii*,* B. coagulans*,* B. ginsengihumi*,* B. firmus*,* B. aquimaris*,* B. sporothermodurans*,* B. smithii*,* B. acidicola*, and *B. sporothermodurans* (38.13%, 36.70%, 47.29%, 35.85%, 41.45%, 44.57%, 35.65%, 40.75%, 39.39%, and 37.27%, respectively) (Table 6). The protein content of *B. kwashiorkori* (2,572) is lower than those of *B. alveayuensis*,* B. shackletonii*,* B. coagulans*,* B. ginsengihumi*,* B. firmus*,* B. aquimaris*,* B. sporothermodurans*,* B. smithii*,* B. acidicola*, and *B. thermoamylovorans* (6,689, 4,727, 2,971, 3,832, 4,922, 4,432, 4,211, 3,619, 4,876, and 3,441, respectively) (Table 6). However, the distribution of genes into COG categories is similar in all compared genomes (Figure 7). In addition, AGIOS values ranged from 54.77% to 67.06% among the *Bacillus* species compared (Table 7). The range of AGIOS varied from 54.77% to 65.50% between *B. kwashiorkori* and other compared *Bacillus* species (Table 7). Moreover, *B. kwashiorkori* shares 455, 500, 340, 375, 541, 490, 461, 283, 451, and 476 orthologous genes with *B. alveayuensis*,* B. shackletonii*,* B. coagulans*,* B. ginsengihumi*,* B. firmus*,* B. aquimaris*,* B. sporothermodurans*,* B. smithii*,* B. acidicola*, and *B. thermoamylovorans*, respectively (Table 7). Of the species with standing in nomenclature, ANI values ranged from 66.46% between *B. coagulans* and *B. aquimaris* to 72.53% between *B. sporothermodurans* and *Bacillus shackletonii*. When comparing *B. kwashiorkori* to other species, the ANI value ranged from 66.74% between *B. kwashiorkori* and *B. coagulans* to 69.92% between *B. kwashiorkori* and *B. thermoamylovorans* (Table 8). The low ANI values confirmed it as a new species because ANI values bigger than 95 indicated that strains belong to the same species (Konstantinidis, Ramette, & Tiedje, 2006). Finally, digital DNA–DNA hybridization (dDDH) estimation of the strain SIT6^T^ against the compared genomes confirmed its new species status, as it ranges between 18.6 and 38.3 (below the cutoff of 70%).

**Table 6:** Genomic comparison of *Bacillus kwashiorkori* with other *Bacillus* spp

Species	Strain	Genome accession number	Genome size (Mb)	GC (%)	Gene content
*B. kwashiorkori*	SIT6^T^	CTDX00000000	2.78	35.19	2,572
*B. alveayuensis*	TM1^T^	JYCE00000000	6.70	38.13	6,689
*B. shackletonii*	LMG 18435^T^	LJJC00000000	5.29	36.70	4,727
*B. coagulans*	2–6^T^	CP003056	3.07	47.29	2,971
*B. ginsengihumi*	Gsoil 114^T^	JAGM00000000	3.92	35.85	3,832
*B. firmus*	IAM 12464^T^	BCUY00000000	4.97	41.45	4,922
*B. aquimaris*	TF‐12^T^	LQXM00000000	4.42	44.57	4,432
*B. sporothermodurans*	M215^T^	LQYN00000000	4.04	35.65	4,211
*B. smithii*	NRRL NRS‐173^T^	BCVY00000000	3.38	40.75	3,619
*B. acidicola*	105‐2^T^	LWJG00000000	5.13	39.39	4,876
*B. thermoamylovorans*	DKP^T^	CCRF00000000	3.70	37.27	3,441

**Table 7:** Number of orthologous proteins shared between genomes (upper right triangle), average percentage similarity of nucleotides corresponding to orthologous protein shared between genomes (lower left triangle), and number of proteins per genome (bold numbers)

	*B. kwashiorkori*	*B. firmus*	*B. shackletonii*	*B. smithii*	*B. aquimaris*	*B. thermoamylovorans*	*B. coagulans*	*B. alveayuensis*	*B. sporothermodurans*	*B. acidicola*	*B. ginsengihumi*
*B. kwashiorkori*	**2,572**	541	500	283	490	476	340	455	461	451	375
*B. firmus*	56.84	**4,922**	928	552	984	642	529	839	837	887	700
*B. shackletonii*	59.33	56.57	**4,727**	592	905	656	628	736	1026	1073	838
*B. smithii*	55.15	59.30	55.92	**3,619**	510	457	466	525	530	603	616
*B. aquimaris*	62.32	58.30	55.88	56.28	**4,432**	574	508	755	779	848	640
*B. thermoamylovorans*	58.24	58.30	56.48	60.21	55.62	**3,441**	526	583	574	577	596
*B. coagulans*	54.77	58.14	54.96	59.33	56.18	58.45	**2,971**	484	553	616	676
*B. alveayuensis*	65.14	57.97	57.07	57.05	63.75	57.46	56.32	**6,689**	639	693	604
*B. sporothermodurans*	58.97	57.07	60.75	58.46	57.02	59.13	55.77	58.53	**4,211**	871	718
*B. acidicola*	63.97	57.72	58.77	57.15	63.91	57.09	57.12	63.93	59.39	**4876**	862
*B. ginsengihumi*	65.50	57.61	59.44	57.66	63.01	58.01	58.03	65.22	60.56	67.06	**3832**

**Table 8:** Average nucleotide identity (ANI) pairwise comparisons among sequenced species in the *Bacillus* genus

	*B. kwashiorkori*	*B. firmus*	*B. shackletonii*	*B. smithii*	*B. aquimaris*	*B. thermoamylovorans*	*B. coagulans*	*B. alveayuensis*	*B. sporothermodurans*	*B. acidicola*	*B. ginsengihumi*
*B. kwashiorkori*	100	68.67	68.52	67.94	67.41	69.92	66.74	68.17	68.67	67.64	68.28
*B. firmus*		100	68.07	67.68	68.02	66.74	67.52	67.90	68.75	68.70	67.43
*B. shackletonii*			100	68.67	68.69	67.68	67.95	68.24	72.53	70.61	70.06
*B. smithii*				100	67.95	67.95	68.52	68.91	68.41	68.41	68.50
*B. aquimaris*					100	66.77	66.46	67.96	68.88	68.31	68.17
*B. thermoamylovorans*						100	67.87	67.81	68.16	67.01	67.80
*B. coagulans*							100	66.84	67.69	68.46	69.39
*B. alveayuensis*								100	68.44	68.34	68.33
*B. sporothermodurans*									100	69.87	70.47
*B. acidicola*										100	69.06
*B. ginsengihumi*											100

ANI values are in percentages. Strains with ANI values over 95% are considered to belong to the same species.

## 4 CONCLUSION

Based on the phenotypic properties (Table 2), phylogenetic tree (Figure 1), MALDI‐TOF analyses (Figure 3), and genomic comparison (taxonogenomics [Table 6 and Table 7] and GGDC results), we propose the creation of *B. kwashiorkori* sp. nov. represented by the strain SIT6^T^.

### 4.1 Description of *B. kwashiorkori* sp. nov

*Bacillus kwashiorkori* (kwa.shi.or.ko'ri. L. adj. masc., in reference to Kwashiorkor) species are gram‐positive, facultative aerobic, short rods, 1.8–5.9 μm in size, and motile by means of peritrichous flagella and sporulating. Colonies are creamy white, circular, 4–5 mm in diameter after cultivation at 37°C for 24 hr on 5% sheep blood‐enriched Colombia agar. Growth occurs at temperatures in the range of 25–56°C (optimally at 37°C). It is catalase and oxidase positive. Concerning the biochemical characteristics, the API 50CH strip showed positive reactions for d‐glucose, d‐fructose, d‐mannose, arbutin, esculin ferric citrate, salicin, d‐maltose, saccharose, d‐trehalose, melezitose, d‐raffinose, and amidon. Negative reactions were recorded for glycerol, erythritol, d‐arabinose, l‐arabinose, d‐ribose, d‐xylose, l‐xylose, d‐adonitol, methyl‐β‐D‐xylopyranoside, d‐galactose, l‐sorbose, l‐rhamnose, dulcitol, inositol, d‐mannitol, d‐sorbitol, methyl‐α‐D‐mannopyranoside, methyl‐α‐D glucopyranoside, *N*‐acetyl‐glucosamine, d‐cellobiose, d‐trehalose, inulin, glycogen, xylitol, gentiobiose, d‐turanose, d‐lyxose, d‐tagatose, d‐fucose, l‐fucose, d‐arabitol, l‐arabitol, potassium gluconate, potassium 2‐ketogluconate, and potassium 5‐ketogluconate.

Using API 20NE, a positive reaction was obtained for d‐maltose, d‐glucose, d‐mannitol, and esculin ferric citrate. But potassium nitrate, l‐tryptophan, l‐arginine, urea, nitrophenyl β‐d‐galactopyranoside, l‐arabinose, *N*‐acetyl‐glucosamine, potassium gluconate, capric acid, adipic acid, malic acid, trisodium citrate, and phenylacetic acid were not assimilated.

API ZYM showed positive reactions for alkaline phosphatase, esterase (C4), cystine aminopeptidase, chymotrypsin, acid phosphatase, phosphoamidase, galactosidase, and mannosidase, but negative results for esterase lipase (C8), lipase (C14), leucine aminopeptidase, valine aminopeptidase, trypsin, α‐glucuronidase, glucosaminidase, and α‐fucosidase.

The major fatty acids are saturated species (15:0 iso, 16:0 and 17:0 anteiso). Strain SIT6^T^ was resistant to metronidazole, but susceptible to imipenem, doxycycline, rifampicin, vancomycin, amoxicillin, ceftriaxone, gentamicin, trimethoprim/sulfamethoxazole, erythromycin, ciprofloxacin, and gentamicin.

The G+C content of the genome is 35.19%. The 16S rRNA gene sequence and whole‐genome shotgun sequence of *B. kwashiorkori* SIT6^T^ are deposited in GenBank under accession numbers LK985393 and CTDX00000000, respectively. The strain SIT6^T^ (=CSUR P2452^T^, =DSM 29059^T^) was isolated from the fecal flora of a Nigerian 4‐month‐old child suffering from acute malnutrition (kwashiorkor). Habitat is the human gut.

## CONFLICTS OF INTEREST

The authors declare no conflicts of interest.

## References

[mbo3535-bib-0501] Albert, R.‐A. , Archambault, J. , Rosselló‐Mora, R. , Tindall, B.‐J. , & Matheny, M. (2005). *Bacillus acidicola* sp. nov., a novel mesophilic, acidophilic species isolated from acidic *Sphagnum* peat bogs in Wisconsin. International Journal of Systematic and Evolutionary Microbiology, 55, 2125–2130. 16166720 10.1099/ijs.0.02337-0

[mbo3535-bib-0001] Angelakis, E. , Armougom, F. , Million, M. , & Raoult, D. (2012). The relationship between gut microbiota and weight gain in humans. Future Microbiology, 7, 91–109. 22191449 10.2217/fmb.11.142

[mbo3535-bib-0002] Auch, A. F. , Jan, M. , Klenk, H.‐P. , & Göker, M. (2010). Digital DNA‐DNA hybridization for microbial species delineation by means of genome‐to‐genome sequence comparison. Standards in Genomic Sciences, 2, 117. 21304684 10.4056/sigs.531120 PMC3035253

[mbo3535-bib-0003] Aziz, R. K. , Bartels, D. , Best, A. A. , DeJongh, M. , Disz, T. , Edwards, R. A. , … Osterman A. L. (2008). The RAST server: Rapid annotations using subsystems technology. BMC Genomics, 9, 75. 18261238 10.1186/1471-2164-9-75 PMC2265698

[mbo3535-bib-0004] Bae, S. S. , Lee, J.‐H. , & Kim, S.‐J. (2005). *Bacillus alveayuensis* sp. nov., a thermophilic bacterium isolated from deep‐sea sediments of the Ayu Trough. International Journal of Systematic and Evolutionary Microbiology, 55, 1211–1215. 15879257 10.1099/ijs.0.63424-0

[mbo3535-bib-0005] Bittar, F. , Keita, M. B. , Lagier, J.‐C. , Peeters, M. , Delaporte, E. , & Raoult, D. (2014). *Gorilla gorilla gorilla* gut: A potential reservoir of pathogenic bacteria as revealed using culturomics and molecular tools. Scientific Report, 4, 7174. 10.1038/srep07174 PMC4241516 25417711

[mbo3535-bib-0006] Carver, T. , Harris, S. R. , Berriman, M. , Parkhill, J. , & McQuillan, J. A. . (2012). Artemis: An integrated platform for visualization and analysis of high‐throughput sequence‐based experimental data. Bioinformatics (Oxford, England), 28, 464–469. 22199388 10.1093/bioinformatics/btr703 PMC3278759

[mbo3535-bib-0007] Carver, T. , Thomson, N. , Bleasby, A. , Berriman, M. , & Parkhill, J. (2009). DNAPlotter: Circular and linear interactive genome visualization. Bioinformatics, 25, 119–120. 18990721 10.1093/bioinformatics/btn578 PMC2612626

[mbo3535-bib-0008] Chen, J. , He, X. , & Huang, J. (2014). Diet effects in gut microbiome and obesity. Journal of food science, 79, 442–451. 10.1111/1750-3841.12397 24621052

[mbo3535-bib-0009] Combet‐Blanc, Y. , Ollivier, B. , Streicher, C. , Patel, B. K. C. , Dwivedi, P. P. , Pot, B. , … Garcia, J.‐L. (1995). *Bacillus thermoamylovorans* sp. nov., a moderately thermophilic and amylolytic bacterium. International Journal of Systematic and Evolutionary Microbiology, 45, 9–16. 10.1099/00207713-45-1-9 7857812

[mbo3535-bib-0010] Coorevits, A. , Logan, N. A. , Dinsdale, A. E. , Halket, G. , Scheldeman, P. , Heyndrickx, M. , … De Vos, P. (2011). *Bacillus thermolactis* sp. nov., isolated from dairy farms, and emended description of *Bacillus thermoamylovorans* . International Journal of Systematic and Evolutionary Microbiology, 61, 1954–1961. 20833876 10.1099/ijs.0.024240-0

[mbo3535-bib-0011] Darling, A.‐C.‐E. , Mau, B. , Blattner, F.‐R. , & Perna, N.‐T. (2004). Mauve: Multiple alignment of conserved genomic sequence with rearrangements. Genome Research, 14, 1394–1403. 15231754 10.1101/gr.2289704 PMC442156

[mbo3535-bib-0012] De Clerck, E. , Rodriguez‐Diaz, M. , Forsyth, G. , Lebbe, L. , Logan, N. A. , & DeVos, P. (2004). Polyphasic characterization of *Bacillus coagulans* strains, illustrating heterogeneity within this species, and emended description of the species. Systematic and Applied Microbiology, 27, 50–60. 15053321 10.1078/0723-2020-00250

[mbo3535-bib-0013] Dione, N. , Sankar, S. A. , Lagier, J.‐C. , Khelaifia, S. , Michele, C. , Armstrong, N. , … Fournier, P.‐E. (2016). Genome sequence and description of *Anaerosalibacter massiliensis* sp. nov. New Microbes and new Infections, 10, 66–76. 26937282 10.1016/j.nmni.2016.01.002 PMC4753391

[mbo3535-bib-0014] Heyndrickx, M. , Coorevits, A. , Scheldeman, P. , Lebbe, L. , Schumann, P. , Rodríguez‐Diaz, M. , … De Vos, P. (2012). Emended descriptions of *Bacillus sporothermodurans* and *Bacillus oleronius* with the inclusion of dairy farm isolates of both species. International Journal of Systematic and Evolutionary Microbiology, 62, 307–314. 21398506 10.1099/ijs.0.026740-0

[mbo3535-bib-0015] Kimura, M. (1980). A simple method for estimating evolutionary rates of base substitutions through comparative studies of nucleotide sequences. Journal of Molecular Evolution, 16, 111–120. 7463489 10.1007/BF01731581

[mbo3535-bib-0016] Kokcha, S. , Ramasamy, D. , Lagier, J.‐C. , Robert, C. , Raoult, D. , & Fournier, P.‐E. (2012). Non‐contiguous finished genome sequence and description of *Brevibacterium senegalense* sp. nov. Standards in Genomic Sciences, 7, 233–245. 23408786 10.4056/sigs.3256677 PMC3569389

[mbo3535-bib-0017] Konstantinidis, K. , Ramette, A. , & Tiedje, J.‐M. (2006). The bacterial species definition in the genomic era. Philosophical transactions Royal Society B Biological Sciences, 361, 1929–1940. 10.1098/rstb.2006.1920 PMC1764935 17062412

[mbo3535-bib-0018] Krogh, A. , Larsson, B. , von Heijne, G. , & Sonnhammer, E. L. (2001). Predicting transmembrane protein topology with a hidden Markov model: Application to complete genomes. Journal of Molecular Biology, 305, 567–580. 11152613 10.1006/jmbi.2000.4315

[mbo3535-bib-0019] Kumar, S. , Stecher, G. , & Tamura, K. (2016). MEGA7: Molecular evolutionary genetics analysis version 7.0 for bigger datasets. Molecular Biology and Evolution, 33, 1870–1874. 27004904 10.1093/molbev/msw054 PMC8210823

[mbo3535-bib-0020] Lagier, J.‐C. , Armougom, F. , Million, M. , Hugon, P. , Pagnier, I. , Robert, C. , … Raoult, D. (2012). Microbial culturomics: Paradigm shift in the human gut microbiome study. Clinical Microbiology and Infection, 18, 1185–1193. 23033984 10.1111/1469-0691.12023

[mbo3535-bib-0021] Lagier, J.‐C. , Elkarkouri, K. , Rivet, R. , Couderc, C. , & Raoult, D. (2013). Non contiguous‐finished genome sequence and description of *Senegalemassilia anaerobia* gen. nov., sp. nov. Standards in Genomic Sciences, 7, 343–356. 24019984 10.4056/sigs.3246665 PMC3764928

[mbo3535-bib-0022] Lagier, J.‐C. , Khelaifia, S. , Alou, M.‐T. , Ndongo, S. , Dione, N. , Hugon, P. , … Raoult, D. (2016). Culture of previously uncultured members of the human gut microbiota by culturomics. Nature Microbiology, 1, 16203. 10.1038/nmicrobiol.2016.203 PMC12094094 27819657

[mbo3535-bib-0023] Lechner, M. , Findeiss, S. , Steiner, L. , Marz, M. , Stadler, P. F. , & Prohaska, S. J. (2011). Proteinortho: Detection of (co‐)orthologs in large‐scale analysis. BMC Bioinformatics, 12, 124. 21526987 10.1186/1471-2105-12-124 PMC3114741

[mbo3535-bib-0024] Matuschek, E. , Brown, D.‐F.‐J. , & Kahlmeter, G. (2014). Development of the EUCAST disk diffusion antimicrobial susceptibility testing method and its implementation in routine microbiology laboratories. Clinical Microbiology and Infection, 20, O255–O266. 24131428 10.1111/1469-0691.12373

[mbo3535-bib-0025] Meier‐Kolthoff, J.‐P. , Auch, A.‐F. , Klenk, H.‐P. , & Göker, M. (2013). Genome sequence‐based species delimitation with confidence intervals and improved distance functions. BMC Bioinformatics, 14, 60. 23432962 10.1186/1471-2105-14-60 PMC3665452

[mbo3535-bib-0026] Morel, A.‐S. , Dubourg, G. , Prudent, E. , Edouard, S. , Gouriet, F. , Casalta, J.‐P. , … Raoult, D. (2015). Complementarity between targeted real‐time specific PCR and conventional broad‐range 16S rDNA PCR in the syndrome‐driven diagnosis of infectious diseases. European Journal of Clinical Microbiology & Infectious Diseases, 34, 561–570. 25348607 10.1007/s10096-014-2263-z

[mbo3535-bib-0027] Moreno‐Indias, I. , Cardona, F. , Tinahones, F. J. , & Queipo‐Ortuño, M. I. (2014). Impact of the gut microbiota on the development of obesity and type 2 diabetes mellitus. Frontiers in microbiology, 5, 190. 24808896 10.3389/fmicb.2014.00190 PMC4010744

[mbo3535-bib-0028] Nielsen, H. , Engelbrecht, J. , Brunak, S. , & von Heijne, G. (1997). Identification of prokaryotic and eukaryotic signal peptides and prediction of their cleavage sites. Protein Engineering, 10, 1–6. 10.1093/protein/10.1.1 9051728

[mbo3535-bib-0029] Ouk, K.‐Y. , Chun, J. , Lee, I. , & Park, S.‐C. (2016). OrthoANI: An improved algorithm and software for calculating average nucleotide identity. International Journal of Systematic and Evolutionary Microbiology, 66, 1100–1103. 26585518 10.1099/ijsem.0.000760

[mbo3535-bib-0031] Ramasamy, D. , Mishra, A.‐K. , Lagier, J.‐C. , Padhmanabhan, R. , Rossi, M. , Sentausa, E. , … Fournier, P.‐E. (2014). A polyphasic strategy incorporating genomic data for the taxonomic description of novel bacterial species. International Journal of Systematic and Evolutionary Microbiology, 64, 384–391. 24505076 10.1099/ijs.0.057091-0

[mbo3535-bib-0032] Rossi‐Tamisier, M. , Benamar, S. , Raoult, D. , & Fournier, P.‐E. (2015). Cautionary tale of using 16S rRNA gene sequence similarity values in identification of human‐associated bacterial species. International Journal of Systematic and Evolutionary Microbiology, 65, 1929–1934. 25736410 10.1099/ijs.0.000161

[mbo3535-bib-0033] Sasser, M. (2006). Bacterial Identification by Gas Chromatographic Analysis of Fatty Acids Methyl Esters (GC‐FAME). Technical Note 101. Newark, DE: MIDI Inc.

[mbo3535-bib-0034] Seck, E. , Sankar, S. A. , Khelaifia, S. , Croce, O. , Robert, C. , Couderc, C. , … Fournier, P. (2016). Non contiguous‐finished genome sequence and description of *Planococcus massiliensis* sp. nov., a moderately halophilic bacterium isolated from the human gut. New Microbes and New Infections, 10, 36–46. 27257487 10.1016/j.nmni.2015.12.006 PMC4877603

[mbo3535-bib-0035] Seng, P. , Abat, C. , Rolain, M. , Colson, P. , Lagier, J. , & Gouriet, F. (2013). Laboratory: Impact of matrix‐assisted laser desorption ionization – time of flight mass spectrometry. Journal of clinical microbiology, 51, 2182–2194. 23637301 10.1128/JCM.00492-13 PMC3697718

[mbo3535-bib-0036] Thompson, J.‐D. , Higgins, D.‐G. , & Gibson, T.‐J. (1994). CLUSTAL W: Improving the sensitivity of progressive multiple sequence alignment through sequence weighting, position‐specific gap penalties and weight matrix choice. Nucleic acids research, 22, 4673–4680. 7984417 10.1093/nar/22.22.4673 PMC308517

[mbo3535-bib-0037] Tindall, B.‐J. , Rosselló‐Mora, R. , Busse, H.‐J. , Ludwig, W. , & Kämpfer, P. (2010). Notes on the characterization of prokaryote strains for taxonomic purposes. International Journal of Systematic and Evolutionary Microbiology, 60, 249–266. 19700448 10.1099/ijs.0.016949-0

[mbo3535-bib-0038] Vartoukian, S.‐R. , Palmer, R.‐M. , & Wade, W.‐G. (2010). Strategies for culture of ‘unculturable’ bacteria. FEMS microbiology letters, 309, 1–7. 20487025 10.1111/j.1574-6968.2010.02000.x

[mbo3535-bib-0039] Weisburg, W.‐G. , Barns, S.‐M. , Pelletier, D.‐A. , & Lane, D.‐J. (1991). 16S Ribosomal DNA amplification for phylogenetic study. Journal of bacteriology, 173, 697–703. 1987160 10.1128/jb.173.2.697-703.1991 PMC207061

[mbo3535-bib-0040] Welker, M. , & Moore, E.‐R.‐B. (2011). Applications of whole‐cell matrix‐assisted laser‐desorption/ionization time‐of‐flight mass spectrometry in systematic microbiology. Systematic and applied microbiology, 34, 2–11. 21288677 10.1016/j.syapm.2010.11.013

[mbo3535-bib-0041] Yoon, S.‐H. , Ha, S.‐M. , Kwon, S. , Lim, J. , Kim, Y. , Seo, H. , & Chun, J. (2017). Introducing EzBioCloud: A taxonomically united database of 16S rRNA and whole genome assemblies. International Journal of Systematic and Evolutionary Microbiology, 67, 1613–1617. 28005526 10.1099/ijsem.0.001755 PMC5563544

[mbo3535-bib-0042] Yoon, J.‐H. , Kim, I.‐G. , Kang, K. H. , Oh, T.‐K. , & Park, Y.‐H. (2003). *Bacillus marisflavi* sp. nov. and *Bacillus aquimaris* sp. nov., isolated from sea water of a tidal flat of the Yellow Sea in Korea. International Journal of Systematic and Evolutionary Microbiology, 53, 1297–1303. 13130010 10.1099/ijs.0.02365-0

[mbo3535-bib-0043] Zhou, Y. , Liang, Y. , Lynch, K. H. , Dennis, J. J. , & Wishart, D. S. (2011). PHAST: A fast phage search tool. Nucleic Acids Research, 39, W347–W352. 21672955 10.1093/nar/gkr485 PMC3125810

