# EXPRESSION OF CONCERN: *Bacillus kwashiorkori* sp. nov., A New Bacterial Species Isolated from a Malnourished Child Using Culturomics

**DOI:** 10.1002/mbo3.70244

**Published:** 2026-02-19

## Abstract

**EXPRESSION OF CONCERN:** E. Seck, M. Beye, S.I. Traore, S. Khelaifia, C. Michelle, C. Couderc, S. Brah, P.‐E. Fournier, D. Raoult, and F. Bittar, “*Bacillus kwashiorkori* sp. nov., A New Bacterial Species Isolated from a Malnourished Child Using Culturomics,” *Microbiology Open* 7, no. 1 (2018): e00535, https://doi.org/10.1002/mbo3.535.

This Expression of Concern is for the above article, published online on 27 October 2017 in Wiley Online Library (wileyonlinelibrary.com), and has been issued by John Wiley & Sons Ltd. The Expression of Concern has been agreed due to questions raised about the study's adherence to French legal and ethical requirements for research involving human subjects, including questions regarding proper informed consent for research involving vulnerable populations. The investigation into these concerns is ongoing. Therefore, the journal has decided to issue an Expression of Concern to inform and alert readers.

